# Analysis of protein expression in periodontal pocket tissue: a preliminary study

**DOI:** 10.1186/s12953-015-0089-y

**Published:** 2015-12-30

**Authors:** Emanuela Monari, Aurora Cuoghi, Elisa Bellei, Stefania Bergamini, Andrea Lucchi, Aldo Tomasi, Pierpaolo Cortellini, Davide Zaffe, Carlo Bertoldi

**Affiliations:** Department of Diagnostic, Clinical and Public Health Medicine, University of Modena and Reggio Emilia, Largo del Pozzo, 71-41124 Modena, Italy; Private Practice, Modena, Italy; European Research Group on Periodontology (ERGOPERIO), Berne, Switzerland; Department of Biomedical, Metabolic and Neural Sciences, University of Modena and Reggio Emilia, Modena, Italy; Department of Surgery, Medicine, Dentistry and Morphological Sciences with Transplant Surgery, Oncology and Regenerative Medicine Relevance, University of Modena and Reggio Emilia, Modena, Italy

**Keywords:** Proteome analysis, Periodontitis, Two-dimensional gel electrophoresis, Protein identification, LC-MS/MS

## Abstract

**Background:**

The periodontal disease is caused by a set of inflammatory disorders characterized by periodontal pocket formation that lead to tooth loss if untreated. The proteomic profile and related molecular conditions of pocket tissue in periodontally-affected patients are not reported in literature. To characterize the proteomic profile of periodontally-affected patients, their interproximal periodontal pocket tissue was compared with that of periodontally-healthy patients. Pocket-associated and healthy tissue samples, harvested during surgical therapy, were treated to extract the protein content. Tissues were always collected at sites where no periodontal-pathogenic bacteria were detectable. Proteins were separated using two-dimensional gel electrophoresis and identified by liquid chromatography/mass spectrometry. After identification, four proteins were selected for subsequent Western Blot quantitation both in pathological and healty tissues.

**Results:**

A significant unbalance in protein expression between healthy and pathological sites was recorded. Thirty-two protein spots were overall identified, and four proteins (S100A9, HSPB1, LEG7 and 14-3-3) were selected for Western blot analysis of both periodontally-affected and healthy patients. The four selected proteins resulted over-expressed in periodontal pocket tissue when compared with the corresponding tissue of periodontally-healthy patients. The results of Western blot analysis are congruent with the defensive and the regenerative reaction of injured periodontal tissues.

**Conclusions:**

The proteomic analysis was performed for the first time directly on periodontal pocket tissue. The proteomic network highlighted in this study enhances the understanding of periodontal disease pathogenesis necessary for specific therapeutic strategies setting.

## Background

The periodontal ligament surrounds the tooth root, connects it to its bony socket constituting a fibrous joint named gomphosis. It contains a large variety of cells and tissues, including immune and stem cells [1, 2]. The periodontitis is a set of inflammatory disorders characterized by gingival and periodontal inflammation, periodontal attachment loss, and alveolar bone resorption, following to periodontal pocket development [3–6].

The periodontal-pathogenic microbiome ecosystems are the most proven risk factor of periodontal disease. Pathogens adhere to and grow on the tooth surfaces, and the inappropriate inflammatory response causes the loss of periodontal attachment and alveolar bone, giving rise to the periodontal pocket, the typical expression of periodontitis, that lead to tooth loss, if untreated.

Periodontitis diagnosis is based on clinical assessment only, inspecting the soft gum tissues around the teeth with a probe (i.e. a clinical examination) in order to detect interproximal attach level-loss and periodontal pocket depth, in the absence of a reliable pathogenic check based on appropriate interpretation of inflammation [5, 7, 8].

Recently, the role of cytokines and other protein mediators of inflammation erupted in all their importance [9]. A modern pathogenetic model incorporating gene, protein, and metabolite data into dynamic biological processes is based on a multilevel framework that include disease-initiating and -resolving mechanisms that are regulated by innate and environmental factors [10].

Now, we can describe more effectively the basic elements of a new model of pathogenesis using emerging genomic, proteomic, and metabolomic techniques [10, 11]. Recent progress in tissue isolation, protein separation, quantification and sequence analysis utilizing novel proteomic techniques promises to bring periodontal physiology and pathology into a new era. A list of inflammation-involved proteins, cytokines, matrix expression and cellular proteins in the periodontal tissues is not currently available. Studies on periodontal diseases utilizing proteomic analysis have been performed on saliva or crevicular fluid samples [12–15], peripheral blood [16–18] or periodontal plaque samples [19], but not on the pathologic tissue of the periodontal pocket, which is the key lesion of the periodontal disease.

To overcome the lack of data, studies assessing the proteomic profile of periodontal pocket tissue and evaluating the molecular characteristics of the periodontally affected patient, are needed.

The aim of this work was to compare the proteomic profile of the pathologic interproximal gingival pocket tissue with that of interproximal gingival healthy tissue, obtained from sites where no periodontal-pathogenic bacteria were detectable.

## Results

Fifteen subjects (T, test group), 3 males and 12 females, ranging in age from 20 to 64 years, average 42.82 ± 13.2 (m ± SD), and fifteen periodontally healthy subjects (C, control group), 6 males and 9 females, ranging in age from 19 to 60 years, average 44.90 ± 11.55, fulfilled study requirements (Table 1). T subjects underwent to the periodontal resective treatment, while C subjects underwent to the crown lengthening surgical treatment. All subjects followed a stringent post-operative supportive care program and achieved satisfactory clinical outcomes.

**Table 1:** Systemic and specific adopted inclusion criteria

Systemic Inclusion Criteria	Local Inclusion Criteria
*Absence of relevant medical conditions*: Medical history of good health (particularly ruling out bone disease, uncontrolled or poorly controlled diabetes, unstable or life-threatening conditions, or requiring antibiotic prophylaxis were excluded).	*Defect anatomy*: Presence of at least one intrabony defect in patient with periodontal disease. Excessive gingival display or gingival margin asymmetries required a surgical correction in periodontally-healthy patients
*Smoking status:* Non-smokers and without a story of alcohol abuse.	*Good oral hygiene*: full-mouth plaque score (FMPS) ≤20 %
*Compliance:* only patients showing high levels of compliance (as assessed during the cause-related phase of therapy) were selected.	*Low level of residual infection:* full-mouth bleeding score (FMBS) ≤20 %.
Pregnancy or lactation and underage were excluded.	*Endodontic status:* Experimental teeth had to be vital or properly treated with root canal therapy.

**Fig. 1:**
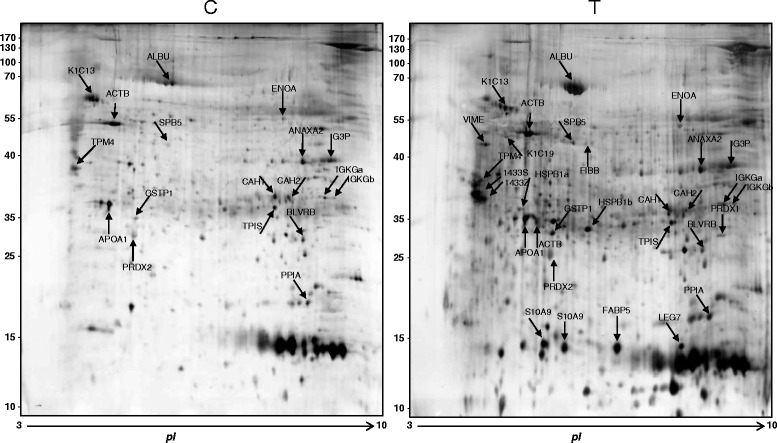
2DE gel of Control and Test tissues. Proteins identified by LC-MS/MS are indicated by Entry name (ID) and pointed by arrows. Control = healthy gingival tissue, Test = periodontal pocket tissues

**Fig. 2:**
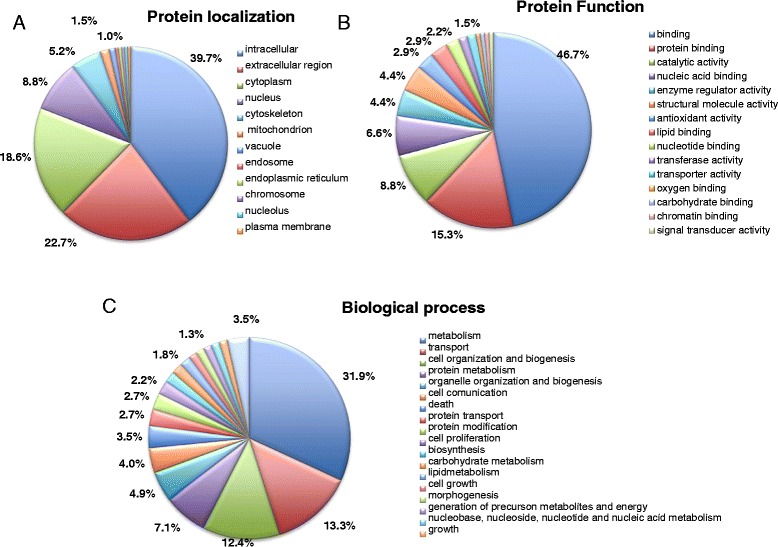
Pie charts describe the distribution of protein localization (**a**), main biological functions (**b**) and biological processes (**c**) of identified proteins of periodontal pocket tissue by GO analysis

2DE (Two-dimensional gel electrophoresis) and LC-MS/MS (Liquid Chromatography-tandem mass spectrometry) analysis were performed for T and C gingival tissue samples. Figure 1 shows representative 2DE gel images of T and C tissues and the 32 protein spots identified. Primary accession number, entry name of UniProt database, MW (Molecular Weight), highest score, matches, sequences and emPAI (Exponentially Modified Protein Abundance Index) for each identified protein are reported in Table 2. Web-based bioinformatics tools (iPROClass and CateGOrize) were employed to investigate all potential localizations, molecular functions and biological processes of the identified proteins (Fig. 2). Intracellular proteins represented the most abundant population (77.3 %). Among them, 18.6 % of protein annotations were recognized as belonging to cytoplasm, 5.6 % to cytoskeleton, and nucleus 8.8 %. Other protein annotations were recognized, with smaller fractions, as belonging to other cellular component such as mitochondrion, vacuole or plasma membrane (Fig. 2a). The reported function of the identified proteins suggests that they are mainly involved in binding (46.8 %), protein binding (15.3 %), and catalytic activity (8.8 %). Other relevant functions are nucleic acid binding (6.6 %), enzyme regulator activity (4.4 %), structural molecule activity (4.4 %), antioxidant activity, lipid binding (2.9 %), nucleotide binding (2.2 %) and transferase activity (1.5 %) (Fig. 2b). In summary, the identified proteins are mainly involved in metabolism (31,9 %), transport (13,3 %) and cell organization and biogenesis (12 %) (Fig. 2c).

**Table 2:** List of identified proteins in periodontal pocket tissues

AC Number^a)^	Entry Name^b)^	Score	Mass (kDa)	pI	Matches^c)^	Significant matches	Sequences^d)^	Significant sequences	emPAI^e)^	Description
P02768	ALBU_HUMAN	5019	71317	5.92	435	310	43	40	6.59	Serum albumin
P13646	K1C13_HUMAN	2745	49900	4.91	247	140	31	23	4.99	Keratin, type I cytoskeletal 13
P19013	K2C4_HUMAN	1509	57649	6.25	176	101	25	18	2.21	Keratin, type II cytoskeletal 4
Q01469	FABP5_HUMAN	1050	15497	6.60	95	44	21	12	16.08	Fatty acid-binding protein, epidermal
P01834	IGKC_HUMAN	829	11773	5.58	65	51	5	5	6.66	Ig kappa chain C region
P01834	IGKC_HUMAN	771	11773	5.58	62	47	5	5	5.11	Ig kappa chain C region
P06733	ENOA_HUMAN	757	47481	7.01	67	40	22	15	2.43	Alpha-enolase
P31947	1433S_HUMAN	592	27871	4.68	88	49	19	12	3.12	14-3-3 protein sigma
P08727	K1C19_HUMAN	492	44079	5.05	51	27	14	9	0.78	Keratin, type I cytoskeletal 19
P00915	CAH1_HUMAN	403	28909	6.59	46	29	8	6	1.39	Carbonic anhydrase 1
P07355	ANXA2_HUMAN	382	38808	7.57	31	17	12	7	0.74	Annexin A2
P04406	G3P_HUMAN	377	36201	5.43	20	19	5	4	0.69	Glyceraldehyde-3-phosphate dehydrogenase
Q06830	PRDX1_HUMAN	358	22324	8.27	53	26	13	6	1.10	Peroxiredoxin-1
P63104	1433Z_HUMAN	317	27899	4.7	43	17	14	8	2.31	14-3-3 protein zeta/delta
P67936	TPM4_HUMAN	282	28619	4.67	51	27	19	14	3.41	Tropomyosin alpha-4 chain
P02647	APOA1_HUMAN	260	30759	5.56	45	22	16	9	5.56	Apolipoprotein A-I
P32119	PRDX2_HUMAN	223	22049	5.66	24	15	9	8	2.10	Peroxiredoxin-2
P06702	S10A9_HUMAN	217	13291	5.71	42	20	8	4	2.94	Protein S100-A9
P47929	LEG7_HUMAN	207	15123	7.03	59	22	7	7	3.74	Galectin-7
P62937	PPIA_HUMAN	197	18229	7.68	11	7	4	3	0.97	Peptidyl-prolyl cis-trans isomerase A
P02675	FIBB_HUMAN	183	55892	8.54	5	3	5	3	0.27	Fibrinogen beta chain
P04792	HSPB1_HUMAN	177	22826	5.98	27	10	9	5	0.83	Heat shock protein beta-1
P60709	ACTB_HUMAN	144	42052	5.29	55	23	13	7	0.67	Actin, cytoplasmic 1
P04792	HSPB1_HUMAN	132	22826	5.98	38	14	7	3	0.69	Heat shock protein beta-1
P09211	GSTP1_HUMAN	124	23569	5.43	6	3	2	1	0.30	Glutathione S-transferase P
P60174	TPIS_HUMAN	118	31057	6.45	17	9	5	4	0.43	Triosephosphate isomerase
P30043	BLVRB_HUMAN	118	22219	7.13	9	5	3	1	0.28	Flavin reductase (NADPH)
P36952	SPB5_HUMAN	80	42530	5.72	4	3	2	2	0.16	Serpin B5
Q9BYX7	ACTBM_HUMAN	76	41989	5.29	2	2	2	2	0.24	Putative beta-actin-like protein 3
P08670	VIME_HUMAN	71	53676	5.06	37	5	10	3	0.20	Vimentin
P06702	S10A9_HUMAN	54	13291	5.71	17	6	3	2	0.83	Protein S100-A9
P00918	CAH2_HUMAN	53	29285	8.15	12	6	3	2	0.33	Carbonic anhydrase 2

^a)^Primary accession number from UniProt database

^b)^Primary entry name from UniProt database

^c)^The number of peptides that matched the identified proteins

^d)^The number of distinct sequences

^e)^Exponentially Modified Protein Abundance Index

**Fig. 3:**
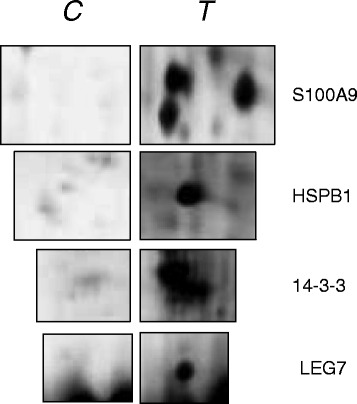
Detailed protein spots, differentially expressed in C and T specimens

PDQuest spot intensity quantification identified four proteins expressed only in the pathological tissue, as shown in Fig. 3, that were selected for subsequent Western blot analysis in both T and C subjects.

**Fig. 4:**
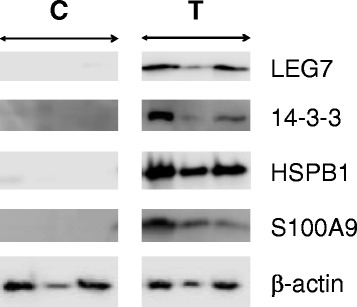
Representative images of Western blot analyses in C and T specimens for LEG7, 14-3-3, HSPB1, S100A9 and β-actin. The β-actin was utilized as reference for protein samples loading and integrity

**Fig. 5:**
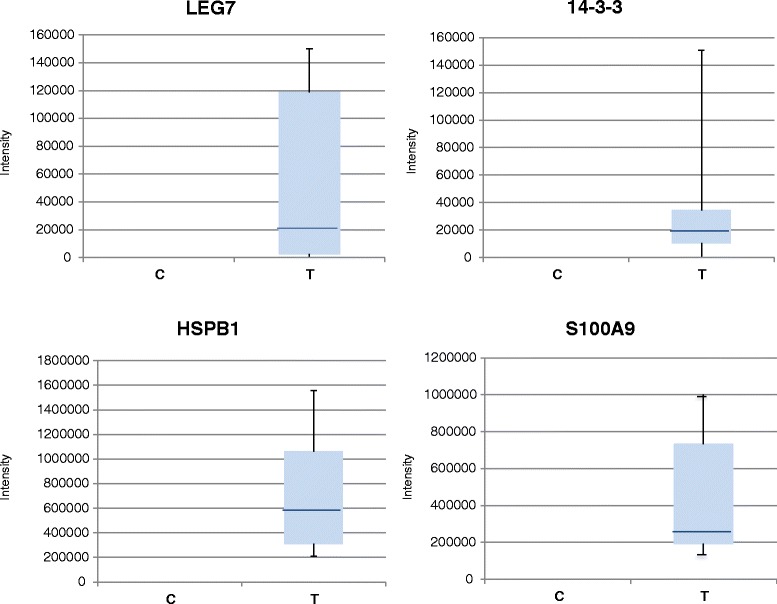
Densitometric analyses of western blot images. Expression level signals are relative to β-actin expression

Representative Western blot images for S100A9, HSPB1, 14-3-3 and LEG7 and β-actin are reported in Fig. 4. Densitometric analyses of Western blot images, normalized to β-actin expression (Fig. 5) confirmed that in C specimens (control tissue), the S100A9, 14-3-3, LEG7 and HSPB1 proteins were not expressed. In pathological tissue (T specimens), the intensity signal for S100A9 and HSPB1 was much higher than the intensity signal of 14-3-3 and LEG7 proteins.

## Discussion

Very little is know on the proteomic analysis of the periodontal pocket tissue [6]. Most proteomic studies have been performed on gingival crevicular fluid, saliva or blood serum, since the non-invasive nature of the collection and availability [20–23]. These studies assume the existence of a direct correlation between the periodontal disease and the biological source to be tested, however there is no direct evidence supporting such assumption. Actually, the periodontal pocket is certainly the anatomo-pathological lesion signifying for an active periodontal disease [7, 8]. Therefore, the periodontal pocket tissue is the only biologic material in which proteomic analysis enables the correct molecular assessment of the periodontal disease. Despite genomic, transcriptomic and proteomic research performed up to now, there are no available biomarkers for periodontitis diagnosis, prognosis and treatment indication giving assistance to the clinician in the disease management. Excluding the studies performed on biologic material different from periodontal tissue, some genomic studies carried out on the gingival tissue showed an association of specific gene polymorphism to periodontitis [24–26].

In our study, proteomics was applied to comparatively analyze the protein content of interproximal tissues of healthy and periodontally affected patients, with the aim to identify differentially expressed proteins. The presence of common periodontopathogenic bacteria was excluded, focusing on disease progression related to the prevailing stimulus, avoiding in this way the possible straining effect caused by periodontopathogenic bacteria on the local protein content. In this way we could discriminate the individual component of the periodontal disease.

The differential expression level observed in 2DE, in pathological and healthy tissues, of 4 proteins, S100A9, HSPB1, LEG7 and 14-3-3, was confirmed by Western blot analysis.

S100A9, is a calcium- or zinc-binding protein involved in the regulation of inflammatory processes and immune response [27], predominantly found as calprotectin (S100A8/S100A9 etherodimer). S100A9 contribute to homeostatic processes that include cytoskeletal rearrangements during trans-endothelial migration of pro-inflammatory phagocytes, binding to receptors as Toll-like receptor 4 and receptor for advanced glycation end products, antimicrobial, oxidant-scavenging and apoptosis-inducing activities [28, 29]. In extracellular fluid, increased levels of S100A9/S100A8 etherodimer were reported in numerous inflammation-associated conditions, such as rheumatoid arthritis, Crohn’s Disease, colorectal cancer and in GCF (Gingival Crevicular Fluid) of patients suffering from gingivitis and periodontitis [30, 31]. The S100A9/S100A8 etherodimer concentration was correlated with periodontal markers of inflammation such as pocket probing depth or gingival bleeding [32, 33]. S100A9 was identified in saliva and proposed as a potential marker to monitor the progression of orthodontic treatment [33]. However, the Authors showed an apparent down-regulation of S100A9 protein, suggesting that this protein could not be involved during bone resorption in orthodontic tooth movement but it was implicated in inflammation. This protein also promotes apoptosis and modulate the inflammatory response in periodontal ligament cells so its down-regulation could suggest a suppression of inflammation [33, 34].

HSPB1 synthesis increases in response to a variety of stresses (e.g. elevated temperatures, heavy metals, toxins, oxidants, bacterial and viral infections) in order to minimize the deleterious consequences of these stimuli and provide the maximal cytoprotective effect [35–39]. In oral tissues, HSPB1 was localized in fibroblasts, odontoblasts, osteoblasts, epithelial cells, endothelial cells of the vascular wall of the dental pulp, and cells of the periodontal ligament [37, 40]. HSPB1 is also a procollagen-binding protein involved in the biosynthesis of type I collagen and major bone extracellular matrix [41]. HSPB1 could be a potential target for the periodontal regeneration process related to cell migration, cytoskeleton maintaining and tissue preservation, also through the modulation of the immune system, and its under-expression induces differentiation abortion, in relation to cell death by apoptosis [40].

Galectin-7 (LEG7) is associated with epithelial cell migration and accelerates the re-epithelialization of wounds. In particular, LEG7 expression contributes to the tissue remodeling processes following tissue damage that involves apoptotic cell death [42]; a defective LEG7 expression could impair the healing processes. LEG7 could function in the maintenance of the normal phenotype of epithelial cell and is activated by a wide range of cellular stresses including UV and γ irradiation. LEG7 protects cell from death by functioning inside the cell and interacting with intracellular proteins. Finally, LEG7 belongs to a protein family promoting healing processes and favor immune responses [43]. HSPB1 and LEG7 would seem to be part of a protein network that play an important role in controlling cell and tissue damage, in moderating the inflammation and destructive immune response. The lack of the previously described multi-protective effect of these proteins, particularly in the presence of inflammation, hinders cells to protect themselves against the cytotoxicity of inflammatory mediators [44], increases their susceptibility to necrotic cell death [45] and probably does not allow an efficient immune response against the noxa [46].

The 14-3-3 protein sigma and zeta/delta are regulatory phosphorserine/threonine binding proteins involved in the control of several cellular events, including cell cycle checkpoint, connective tissue remodeling, apoptosis signaling, Toll-like receptor activation and TNF production during inflammation response [47, 48]. In particular the 14-3-3 sigma induces up-regulation of differentiation, down-regulation of cell proliferation and collagenase induction as matrix metalloproteinase-1 [47, 49, 50]. Wu et al. [12] found decreased 14-3-3 protein sigma in saliva of subjects with generalized aggressive periodontitis, and, analyzing the GCF, Huynh et al. [15] found 2–3 time higher level in gingivitis than in chronic periodontitis.

Our results are congruent with an inflammatory response oriented to defense and regeneration of injured tissues. S100A9, HSPB1, LEG7 and 14-3-3 proteins resulted over-expressed in periodontal pocket tissue when compared with healthy patient analogous tissue. A significant unbalancing in protein expression between healthy and pathological sites was recorded. In a previous study [6] we aimed to compare the interproximal pocket tissue with interproximal tissues at sites with normal probing depth in patients affected from periodontal disease and HSPB1, LEG7 and 14-3-3 proteins resulted significantly under-expressed. Also S100A9 resulted under-expressed, but not significantly.

In all likelihood, patients have to be regarded as affected by periodontal disease because a complex pathologic network (composed by genetic structure involving immunology and inflammatory regulation) at the root of periodontitis even if the periodontal disease is being clinically burnt out [51–54].

A limited number of biomolecular studies have been carried out on the periodontal pocket. Hence, inadequate data are available on the pathognomonic lesion of the periodontal disease. Genes related to apoptosis, antimicrobial humoral response, antigen presentation, regulation of metabolism, signal transduction and angiogenesis were found to be differently expressed in patients with periodontitis and healthy subjects, as found in trascriptomic studies [55, 56]. Moreover, with the aid of microarray technologies, cell communication pathways were shown to be down-regulated in periodontitis-affected tissues, either in cell-to-cell communications at the soft tissue level, or in cell-to-tooth signaling as a consequence of the inflammatory status of the periodontium [55, 56].

It is conjecturable that an high inflammatory background is anyway present in healthy gingival tissues of patients with periodontal disease, and the change of the inflammatory and immunologic processes could have a role in periodontal damage with the hindering of the protecting molecular network when the risk factor challenge induces the periodontal pocket.

The identified proteins play a role in increased cell proliferation, decrease in cell tissue differentiation, impairing of metalloproteinases on connective tissue, organized action monocytes/macrophages (e.g. osteoclasts) and osteoblasts or fibroblast together with enhanced humoral and cell-mediated immunity. All these processes are consistent with a defensive response of the organism that also aims to regenerate the lost tissue after exogenous injury. Furthermore, the phagocytosis of opsonized cells, the alternative way by Toll-like receptors 2 and 4 or the T-cell activation to bacterial LPS could contribute to microbiota clearance along with the presence of chemo attractant molecules in the recruitment of immune cells. During these phases, an increasing cell migration could also play an important role in the recovery of damaged tissue as periodontal ligament or bone. It is also to consider that HSPs, whose synthesis is increased in response to a variety of stresses, and S100A9 play an important role in controlling cell and tissue damage, and in moderating inflammation and destructive immune response. HSPB1 and S100A9 resulted significantly increased, in all the tested pathological sites, while LEG7 and 14-3-3 proteins, although not expressed in normal tissue, exhibited fluctuation in expression level in periodontal lesions. These results need further studies to understand if the lack in expression of these proteins may worsen the tissue damage.

## Conclusions

Most proteomic studies have been performed on different biological samples but not on periodontal pocket tissue that is the anatomo-pathological lesion signifying for the clinical diagnosis of the periodontal disease. Therefore, the periodontal pocket tissue is the only biologic material in which proteomic analysis enables the correct molecular assessment of this pathology. The establishment of a robust proteomic expression profile database for periodontal pathology would be highly desirable both to understand the pathogenesis and for periodontitis therapeutic strategies [13]. This is the first work that compares the proteomic content of periodontal pocket lesions and healthy gingival tissue of healthy subjects. The results of this study pointed out a network of proteins, differently expressed in the pathological tissue compared with the healthy one and contribute to the establishment of a proteomic expression profile database for periodontal pathology. These data are highly desirable both to understand the pathogenesis and for periodontitis therapeutic strategies, though further population studies are required to correlate the proteomic data with clinical data, as disease progression and aggressiveness.

## Methods

### Sample collection

Systemic healthy subjects, with and without periodontal chronic disease, examined and treated in a private dental office, were enrolled in this study, according to the protocol described in Table 1. Italian law does not require any ethical committee authorization for clinical trials performed in private dental offices, while such authorization is required for public dental health centers (DM 18/3/1998 published in the Official Gazette, GU n. 122 of 28-05-1998). Therefore, for the purposes of this study all enrolled subjects signed an informed consent form detailing the study procedures. The research was carried out in full accordance with the ethical principles of the WMA Helsinki Declaration [57].

Fifteen periodontally-healthy patients underwent crown lengthening surgery, where gingiva in excess or gingival margin asymmetries required a surgical correction [58, 59]. Periodontally-affected subjects presented at least a shallow intrabony defect suitable for treatment by osseous resective surgery.

Samples for microbial analysis were obtained from patients immediately before surgery, in order to exclude periodontopathogenic bacteria. The samples were analyzed by PCR-RT technique (GABA International AG, Lorrach, Germany), to confirm the absence of * Actinobacillus actinomycetemcomitans*, *Porphyromonas gingivalis*, *Tannerella forsythensis*, *Treponema denticola*, *Fusobacterium nucleatum ssp*, and *Prevotella intermedia*.

The surgery was performed in both periodontally-healthy and -affected patients following the completion of a preliminary cause-related treatment required for the surgical approach and having reached a full-mouth plaque score and full-mouth bleeding score lower than 20 %.

Tissue specimens of interproximal healthy tissues were harvested in fifteen periodontally-healthy patients (C, control) at sites with normal probing depth [58–60]. Tissue specimens of interproximal pocket-associated tissue were harvested in subjects affected by chronic periodontitis (T, test). The harvested tissues were immediately frozen at −80 °C, for proteomic analyses.

### Sample preparation for proteomic analysis

Tissue samples were ground in small pieces in a mortar with liquid nitrogen and collected in tubes. Tissue lysate was performed incubating the samples for 1 h with a buffer containing 7 M urea, 2 M thiourea, 3 % CHAPS, 40 mM Tris pH 8.3, 1 % ampholytes pH 3–10, protease inhibitors at room temperature. After incubation, tissues were further disrupted with an ultrasonic homogenizer (Sonoplus HD 2070, Bandelin electronic, Germany), and centrifuged at 10,000 x g for 10 min at +4 °C. Supernatant was precipitated by the addition of cold acetone (dilution ratio 1:12 vol/vol) and incubated at −20 °C overnight. After centrifugation at 14,000 x g for 15 min at +4 °C, the pellet was re-suspended and the protein concentration was determined according to the Bradford method. Three pooled samples, both for pathological and healthy gingival tissues, were obtained by mixing equal protein amount of 5 different subjects and were analyzed in duplicate.

### 2-Dimension electrophoresis

Tissue extracted proteins were separated by 2DE following a protocol previously described [61]. Briefly, for the first dimension, 80 μg of total proteins were loaded onto IPG strips, 17 cm long, pH range 3–10 (ReadyStripTM, Bio-Rad, USA). Afterwards, the second-dimension separation was carried out at 10 °C using 12 % polyacrylamide gels. Between these two separation steps, strips were reduced with 1 % DTT, and later alkylated with 2.5 % iodoacetamide in an equilibration buffer (6 M urea, 1 % DTT, 50 mM Tris–HCl pH 8.8, 30 % glycerol, 2 % SDS). After 2-DE, the protein spots in the gels were visualized following a silver nitrate staining protocol, as previously described [62]. The silver-stained gel images were acquired using a GS-800 Calibrated Densitometer (Bio-Rad, USA) and analysed with the PDQuest 2-D Image software program, version 7.3.1 (Bio-Rad, USA).

### Protein identification by LC-MS/MS analysis

Protein spots excised manually from the gels were subjected to the “in-gel” tryptic digestion as previously described [63]. Dried samples were then re-suspended in 97 % Water/3 % ACN added of 1 % formic acid (Buffer A) and analyzed by a Nano LC-CHIP-MS system, consisting of the Agilent 6520 ESI-Q-TOF, coupled with a 1200 Nano HPLC-Chip microfluidic device (Agilent Technologies Inc., USA). Four microliters of each sample were loaded into the system and transported to the Chip enrichment column (Zorbax C18, 4 mm x 5 μm i.d., Agilent Technologies) by a capillary pump, with a loading flow of 4 μL/min, using 95 % ACN/5 % water added of 0.1 % formic acid (buffer B) as mobile phase. Nitrogen was used as the nebulizing gas. A separation column (Zorbax C18, 43 mm x 75 μm i.d., Agilent Technologies), at flow rate of 0.4 μL/min, was used for peptide separation. Protein-identification peak lists were generated using MASCOT search engine (http://mascot.cigs.unimo.it/mascot) against the UniProt Knowledgebase database (UniProt.org), specifying the following parameters: Homo sapiens taxonomy, parent ion tolerance ± 20 ppm, MS/MS error tolerance ± 0.12 Da, alkylated cysteine as fixed modification and oxidized methionine as variable modification, and two potential missed trypsin cleavages. Proteins that were identified with at least 2 or more significant peptides sequences and with the highest score hits among MASCOT search results. “High-scoring” corresponded to proteins that were above the significant threshold in Mascot searches (5 % probability of false match for each proteins above this score).

### Protein functional analysis

The protein functional analysis and classification of identified proteins were performed using the iPROClass integrated database (http://pir.georgetown.edu/pirwww/dbinfo/iproclass.shtml accessed on March, 2015) for protein annotation and GO Terms Classifications Counter (CateGOrize, http://www.animalgenome.org/bioinfo/tools/countgo/ accessed on March, 2015) for clusterization according to the Gene Ontology (GO) hierarchy.

### Western blot analysis

For Western blotting experiments, 3 μg of gingival tissue protein extracts were solubilized in Laemmli’s buffer 1X and denatured for 5 min at 95 °C. Protein content was resolved by 12 % or 15 % SDS-PAGE and the proteins were transferred onto a nitrocellulose membrane by electroblotting. Membranes were blocked at 4 °C overnight with 5 % non-fat dry milk in PBS containing 0.1 % Tween 20. The membranes were incubated with the relevant antibodies for 2 h (1:1000 dilution), washed, and incubated with HRP coniugated Polyclonal Goat Anti-rabbit secondary antibody (1:2000 dilution, DakoCytomation, Denmark) for 1 h. The proteins were visualized using WesternSure Premium Chemiluminescent substrate (LI-COR, USA) and C-Digit Blot scanner (LI-COR, USA) according to the manufacturer’s instructions. Acquired images were analyzed and compared using Image Studio Software 4.0.21 (LI-COR, USA). The sources of primary antibodies were as follows: anti-galectin 7 (ab10482), anti-HspB1 (ab1426) and anti-14-3-3 all isoforms (ab9063) were from Abcam (UK); anti- S100A9 (PA1-46489) was from Thermo scientific (USA). β-actin (ab8227, Abcam UK) was used for normalization of western blot analysis.

## Abbreviations

2DE: Two-dimensional electrophoresis
LC-MS/MS: Liquid Chromatography- Mass/Mass spectrometry
MW: Molecular weight
emPAI: Exponentially modified protein abundance index
HSPB1: Heat shock protein ß-1
LEG7: Galectin-7
1433S: 14-3-3 Protein sigma
1433Z: 14-3-3 protein zeta/delta
S100A9: Protein S100-A9
GCF: Gingival Crevicular Fluid
CHAPS: 3-[(3-Cholamidopropyl) dimethylammonio]-1-propanesulfonate
DTT: Dithiothreitol
ACN: Acetonitrile
ESI-Q-TOF: Electrospray ionization quadrupole time-of-flight mass
SDS-PAGE: Sodium dodecyl sulfate polyacrylamide gel electrophoresis
HRP: Horseradish peroxidase
